# Are There Biomimetic Lessons from Genetic Regulatory Networks for Developing a Lunar Industrial Ecology?

**DOI:** 10.3390/biomimetics6030050

**Published:** 2021-08-09

**Authors:** Alex Ellery

**Affiliations:** Department of Mechanical & Aerospace Engineering, Carleton University, 1125 Colonel By Drive, Ottawa, ON K1S 5B6, Canada; aellery@mae.carleton.ca

**Keywords:** genetic regulatory networks, holonic architecture, industrial ecology, manufacturing architectures, in situ resource utilisation

## Abstract

We examine the prospect for employing a bio-inspired architecture for a lunar industrial ecology based on genetic regulatory networks. The lunar industrial ecology resembles a metabolic system in that it comprises multiple chemical processes interlinked through waste recycling. Initially, we examine lessons from factory organisation which have evolved into a bio-inspired concept, the reconfigurable holonic architecture. We then examine genetic regulatory networks and their application in the biological cell cycle. There are numerous subtleties that would be challenging to implement in a lunar industrial ecology but much of the essence of biological circuitry (as implemented in synthetic biology, for example) is captured by traditional electrical engineering design with emphasis on feedforward and feedback loops to implement robustness.

## 1. Introduction

Nature is renowned for its frugality. Biomimetics is the application of lessons from the natural world into the engineered world—although mostly applicable to robotics, there are biological lessons for other engineering applications. We shall examine lessons from biology that point the way in which we may conduct and organise in situ resource utilisation (ISRU) on the moon. Our first priority is to ensure that we engage the moon’s resources in a sustainable manner. ISRU is usually proposed as a means to reduce the costs of human missions. If resources can be extracted locally from the moon, then these resources do not require launch and delivery from Earth. In particular, consumables—specifically water—are of interest because they are a major input to environment control and life support system (ECLSS) and their extraction involves minimum processing. Hence, the high degree of interest directed towards the discovery of water ice at the lunar poles [1]. A human requires a minimum of 4.4 kg of water per day for consumption only (including 2.5 kg for imbibing with the rest as hydrated food but excluding personal and communal hygiene), 0.9 kg of oxygen per day and 0.7 kg of dehydrated food per day. If water was available for consumption and as a source of oxygen, this would save 5.4 kg per astronaut per day (almost 2 tonnes of launched mass per astronaut per year, which equates to a saving of some 40 tonnes of launch propellant). Furthermore, the implementation of closed ecological life support systems (CELSS) which recycle water and oxygen conserves these valuable lunar resources. Current proposals to burn hydrogen/oxygen as propellant/oxidiser for launch and in-space propulsion is not sustainable. We do not regard this as a wise approach to lunar colonisation. Sustainability is premised on ensuring that future generations are not faced with a barren wasteland resulting from reckless exploitation by current generations. Implicit in this definition is the need to plan our ISRU practices over the long term to ensure [2]: (a) we do not consume and waste scarce resources; (b) we employ renewable technologies as far as is feasible; (c) we adopt processes that do not yield toxic material; (d) we minimise waste through recycling loops. To observe this, we need to design a long-term approach to lunar ISRU that adopts the philosophy of Indigenous peoples—exploit that which is abundant and waste nothing. However, ultimately, we must live off the land as much as possible to minimise our reliance on an Earth-based supply chain. We propose a robotic approach to ISRU that implements an industrial infrastructure that supports a wide range of capabilities that can support both robotic and human activities on the moon with minimal supply from Earth.

Our lunar industrial ecology is designed to supply the processed lunar materials required to construct a generic spacecraft (demandite). We discuss the issue of architectural organisation of the lunar industrial ecology by considering architectural lessons from the manufacturing sector in which flexible reconfiguration is a requirement. We then proceed to examine biomimetic approaches based on lessons from metabolic and genetic regulatory networks. We conclude that there are several lessons including the adoption of holonic architectures but the implementation of genetic regulatory networks is likely to be complex to implement. 

## 2. Lunar Industrial Ecology

We have developed a lunar industrial ecology, the industrial ecology being a concept evolved for sustainable terrestrial chemical engineering, as the basis for industrialising the moon in a rational fashion. The basic unit of the lunar industrial ecology is the unit chemical processor. A chemical plant comprises a set of reaction vessels for mediating chemical reactions to produce chemical products. Chemical processes are divided into steps (unit operations) defined by a process that occurs within a single reactor vessel. Unit operation is a basic analytical approach in chemical processing—it involves a physical or chemical transformation of a set of reagents into a set of products, e.g., mixing, separating or distilling, heating or cooling, redox reactions, de(hydrogenation), halogenation, and polymerisation. It typically involves fluid flow, heat transfer and mass transfer, resulting in thermodynamic and mechanical processes. The vessels are typically cylindrical with rounded ends suitable for high pressures or vacuums. Each reactor comprises a unit operation defined with a quantified input feed and a quantified output product. Within a single reactor, the unit operation constitutes the chemical process which converts one compound (the input reagents) into another compound (the output products). The chemical processes may be run in a continuous or batch mode—in either case, catalysts and packed beds that have been poisoned by deposits may have to be regenerated periodically. The mass transfer rate of fluids through a reactor is defined through dimensionless numbers: $Sh=C.Re^{n}Sc^{m}$ where $Sh=\frac{kL}{D}$ = Sherwood number, $Re=\frac{v\rho L}{\mu }$ = Reynolds number, $Sc=\frac{\mu }{\rho D}$ = Schmidt number, C = empirical constant, k = mass transfer coefficient (dimensions of velocity), A = cross sectional area, L = characteristic length, D = mass diffusivity, v = fluid flow velocity, μ = fluid dynamic viscosity and ρ = fluid density. The obvious way to increase mass flow rate is to increase the pressure difference between the input and output ports of the reactor achieved through motorised pumps. Material and energy balances must be analysed based on chemical analysis. Indeed, diffusion equations for momentum, heat and mass transfer all have similar form as Newton’s equation, Fourier’s conduction law and Fick’s law, respectively. Material composition management (MCM) is the core problem in chemical and manufacturing processes [3]. Conservation of mass requires that all material entering the chemical process either accumulates or exits the process as product or waste (material balance). The reactants are injected into the reaction chamber, within which the operating conditions (temperature and pressure) determine the reaction progression. Furthermore, the reaction itself influences those operating conditions which must be measured continuously. The reaction is also determined by the composition of the reagents and their physical properties. The controller must optimise the conditions and reagent flows to maximise the product yield whilst minimising waste. Complexity is introduced by chemical instabilities, uncertain and incomplete measurement data, limited predictive and diagnostic models, and time delays in chemical dynamics which requires intelligent control with process monitoring based on noisy data to deal with event-driven situations. 

An example of a single unit chemical processor is the Metalysis FFC process reactor [4] that is central to the lunar industrial ecology. Our lunar industrial ecology processes lunar minerals and volatiles into our demandite list of desired functional materials based on requirements for a generic robotic spacecraft (Table 1). 

The most important lunar minerals for metal extraction include ilmenite (Fe and Ti), anorthite (Al, Si and Ca) and orthoclase (K and Si). The only material required from Earth is NaCl as a recycled reagent of the ecosystem (it is not consumed). We require Ni-Fe-Co meteoritic material available in lunar craters [5], from which these and other elements (such as W and Se) may be extracted. Lunar volatiles of interest include hydrogen (from water), carbon compounds and small amounts of nitrogen which can be extracted thermally as regolith volatiles and fractionally condensed (sulphur compounds may be treated similarly but may be better sourced from meteoritic FeS). We utilise volatiles only as recycled reagents so that they are not consumed (with the exception of carbon that is the most abundant volatile by mass). The carbon may provide the basis for silicone plastic or oils manufacture for highly restricted applications to minimise its consumption. The adoption of mineral preprocessing with HCl acid permits the Metalysis FFC process to reduce resultant pure metal oxides into metal powder with >99% purity. Some of the pure metal oxides such as Al_2_O_3_, SiO_2_ and CaO have refractory applications. The CaCl_2_ electrolyte which is not consumed but will suffer small losses may be re-supplied as a byproduct of metal extraction though this consumes imported Cl.

**Scheme 1:**
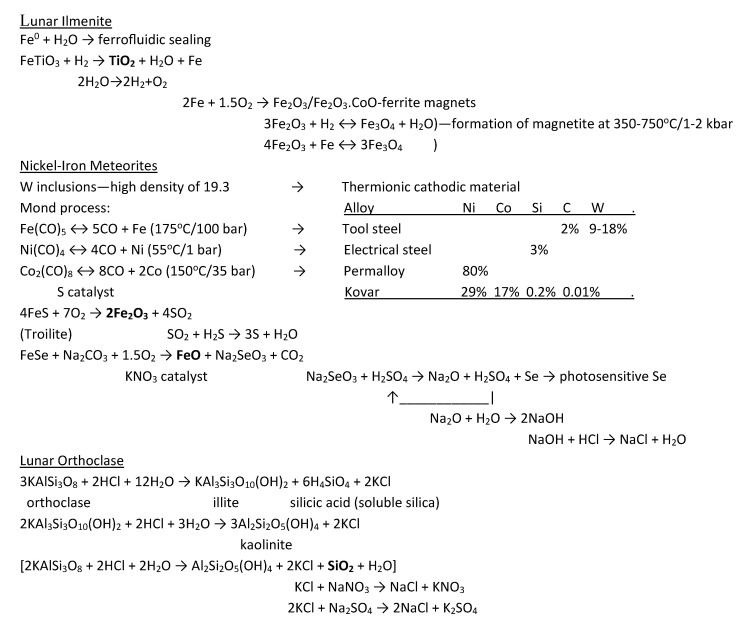

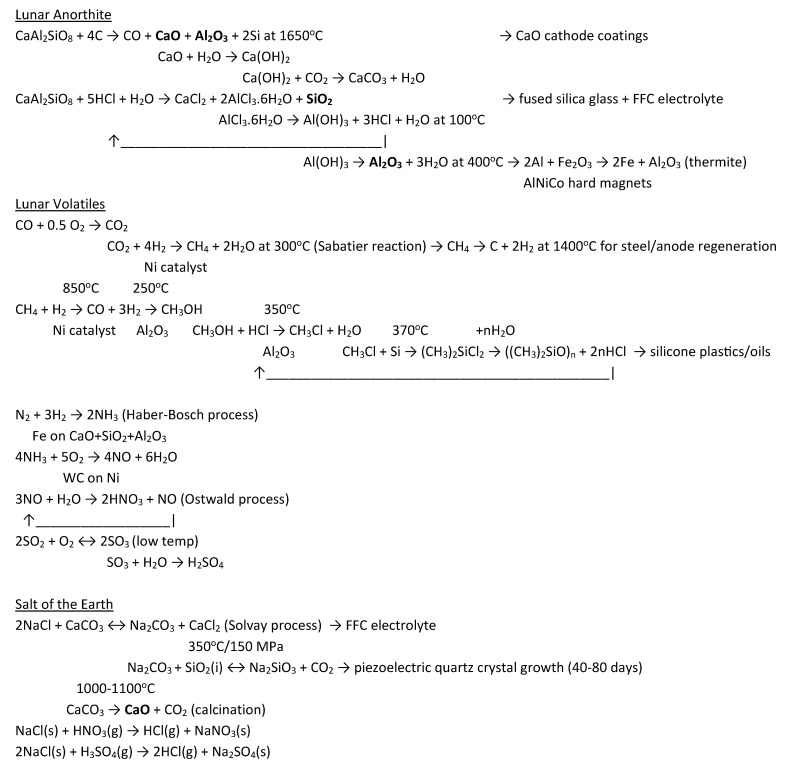
Near closed loop lunar industrial ecology (emboldened materials are pure metal oxides for direct reduction using the Metalysis FFC process). This summarises the detailed sustainable lunar industrial ecology presented in [2]. Inputs to the lunar industrial ecology are energy and lunar raw materials and outputs are processed materials—reagents are recycled.

The lunar industrial ecology (Scheme 1) is an approach to ISRU that is sustainable by linking many different chemical processes together into an ecological system in which the waste of one process becomes the feedstock for another. The lunar industrial ecology constitutes multiple unit chemical processors—this is unique to the lunar environment as most terrestrial chemical processing systems involve only a small number of single throughput processors. On Earth, unit chemical processors are developed for a market in which inputs are processed into useful products with little regard to waste. Terrestrial industrial ecology concepts feed the waste of one unit chemical processor as input into another unit chemical processor in an ad hoc manner driven by economic considerations. Our lunar industrial ecology is designed to form a network of unit chemical processors in which there is minimal waste through nested recycling loops of waste employed as feedstock. The waste generated such as clays is non-toxic and has potential applications in building habitats [6] and in agricultural food production [7]. The lunar industrial ecology essentially constitutes a fan-in to a suite of 3D printing facilities forming the core of a bowtie configuration from which manufactured products fan out [8,9]. 

## 3. Lessons from the Factory

**Figure 1:**
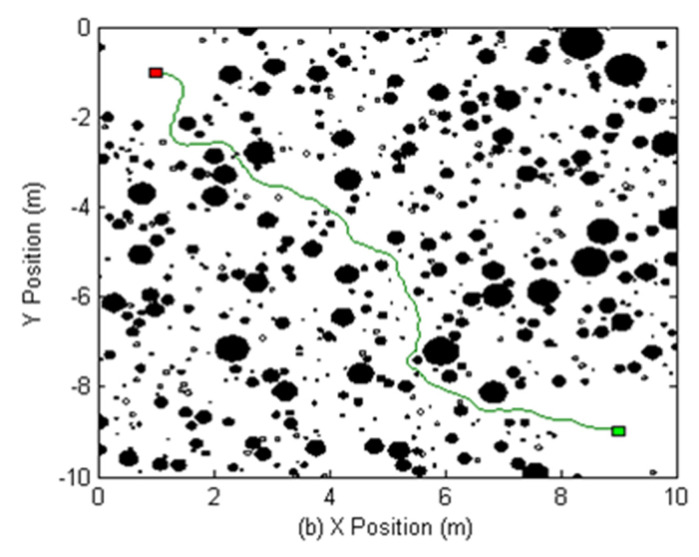
Artificial potential field path through a rockfield (repellors) towards a goal (attractor)—this rockfield models the Mars Viking 2 landing site but appropriate constraints may be crater slopes, slippery locations due to loose soil, etc., which impose constraints on straight line paths between start and goal locations.

As a fully interconnected ecology, it is necessary to ensure that it is reconfigurable to provide flexibility for feeding waste into different processes as required to manufacture different products. Our lunar industrial ecology must be architected to operate in a coordinated fashion to honour recycling loops between processes with maximum efficiency. Terrestrially, it is rare to coordinate and reconfigure unit chemical processors (except through market forces) but it is common in flexible manufacturing systems (FMS) to reconfigure and coordinate different machine units in a single factory for fabricating different products. Lessons may be applied from the manufacturing factory and, indeed, into which the lunar industrial ecology must be integrated as pre-processing to manufacturing. For example, a traditional factory layout arranges machines into functional sections—milling, grinding, drilling, etc. Unfortunately, in such layouts, approximately 95% of total throughput time is spent in transport or queuing for processing. A material flow network connecting modules of machines represents the most efficient production layout [10]. It combines the adaptability of the distributed layout with the high transport efficiency of compact layouts. Physical transport networks must adapt to local conditions. The Zipf inverse distance law quantifies the volume of material N as inversely proportional to the distance D travelled: $N=\frac{k}{D}$. A more sophisticated gravitational model declares that distance travelled is dependent on the strength of attraction imposed by additional factors such as relief, obstacles, and vacancies/queues: $N=k\frac{\left(w_{i}p_{i}-w_{j}p_{j}\right)}{d_{ij}^{a}}$ where *p_i_* = demand at location *i*, *p_j_* = demand at location *j*, *d_ij_* = distance between locations *i* and *j*, *a* = exponent of distance that determines the sharpness of attraction, and *w_i,j_* = weighting factors that quantify other factors such as relief and obstacles. This can be modelled readily by a potential field representation to minimise distance for the transport of material, e.g., [11,12] (Figure 1). Genetic algorithms have been applied to finding a minimum cost machine layout for a factory floor based on interaction forces between different manufacturing activities [13].

An automated factory requires several functions: (i) product specification of complete product; (ii) production planner to schedule and coordinate manufacturing; (iii) parts production—in this case, primarily through 3D printing technologies; (iv) material handling and transport by mobile robots and conveyors; (v) parts assembly by manipulators including jigs; (vi) parts inspection by sensors through self-diagnosis; (vii) computer coordination of the production process. Most industrial processes can be operated without human intervention, the human aspect being reserved primarily for setup, reprogramming and servicing. These can similarly be automated with enhanced autonomy. Setup and servicing require sophisticated manual dexterity which is the preserve of manipulator robotics which has applications to space debris mitigation and on-orbit servicing [14,15]. The axiomatic approach to manufacturing factory design flows down from its top-level functional requirements to the design parameters. There are two axioms of manufacturing [16,17,18]: (i) maintain independence of a minimum number of functional requirements of a product (independence axiom); (ii) minimise information content (cost) of a product consistent with (i) (information axiom). This may be formalised as: {FR} = [A]{DP} where FR = functional requirements vector, DP = design parameters, A = design matrix = diagonal matrix when axiom (i) is observed. A critical aspect is the decomposition of high-level system requirements (what) into low-level components of that system to achieve the requirements (how) [19]. Effective design for manufacturing can reduce manufacturing costs by 80% [20]. The methodology has subsequently been widened to incorporate design for manufacturing and assembly including logistics to minimise production costs [21]. 3D printing is an approach that effectively minimises costs consistent with these principles and indeed offers a more versatile mode of manufacture than subtractive modes. The methodology can be widened to incorporate the (electro)chemical processing of raw materials and the mining chain, i.e., from raw material mining through to final product. 

## 4. Flexible Manufacturing Systems (FMS)

The flexible manufacturing system (FMS) approach provides the ability to reconfigure itself with high flexibility to adapt to manufacturing different products. FMS has been enabled by integration of computer-aided design (CAD), computer-aided manufacturing (CAM), computerised numerically controlled (CNC) machining and robots to collectively form computer integrated manufacturing (CIM) systems [22,23]. CIM is the key element in FMS in which distributed workstations are linked by computer-controlled material handling systems. Material handling processes are an essential part of designing the material flow structure in any distributed manufacturing system. Automation implies a reduction (towards elimination) of human labour with higher throughput at higher quality at lower cost. CIM integrates CNC machines and assembly robots, production control through just-in-time (JIT) manufacture, and transport vehicle control that are part of FMS. The manufacturing cell has a highly automated compact footprint as the basic unit of FMS [24]. FMS comprise multiple cells of robots, CNC machines and material handling linked by a computer network to maximise its autonomous capabilities [25]. It is a multicell system interconnected by self-driving transport vehicles (usually but not necessarily on guide rails) between cells. A cellular manufacturing system is a type of FMS based around groups of machines (a cell) that are specialised for a specific function [26,27]. Cellular manufacturing groups all related activities together into a CNC machining centre tended by a central robot to minimise human intervention. A robot can select a workpiece and emplace it onto a conveyor to transport it to a CNC machine. Another robot within the cell picks up the workpiece and emplaces it into the CNC machine. Finished parts are removed, emplaced onto another conveyor and picked up by another robot for assembly with other parts. Cellular manufacturing reduces work-in-progress allowing a JIT approach [28,29]. Deadlocks can occur when two or more parts require the same resources at the same time. This can invoke a freezing behaviour unless the deadlock is resolved through detection and recovery methods. A typical manufacturing cell includes five functions overseen by a centralised cell supervisor—manufacturing operations, machining planning, workpiece preparation, supplementary operations and inventory stocking [30]. The cell supervisor controls and co-ordinates machine tools, robots, sequencing tasks, production processes, parts and instigates quality control. Only manufacturing operations add value to the workpiece through the input of energy, information and material so all other aspects of manufacture must be minimised. An FMS may be defined as a 7-tuple FMS = {M, B, H, Op, C, Mob, T} where M = set of machines, B = set of buffers, H = set of material handling systems, Op = set of operations for each machine, C = storage capacity for each buffer, Mob = mobility of position range of handling system; T = transportation capacity of handling system. This may be transformed into a manufacturing model P = {G,P} where G = set of manufacturing processes, P = set of finished products. An FMS specification may be transformed automatically into a coloured Petri net model [31]. In the automated lights-out manufacturing system, the intelligent cell provides automated manufacturing, planning and stocking functions with zero manual preparation and supplementary functions. An example of an automated lights-out factory is the Japanese FANUC system of two-armed industrial robots equipped with vision and force/torque sensors that assemble robots on automated production lines. Although, automated production can run unsupervised for weeks, the FANUC system has not yet expanded throughout the manufacturing industry.

The manufacturing cell concept provides a balance between flexibility and efficiency. Mass production maximises efficiency at the expense of flexibility while small lot production of complex systems requires high flexibility at a cost of decreased efficiency. The need is to provide a balance between these two factors which allows frequent customised redesign but high production efficiency. Product variants can be accommodated readily from raw materials to parts to assemblies. This flexibility has several components—organisational flexibility to adapt to changes, machine flexibility to implement different machining operations, material handling flexibility to move different part types efficiently, operational flexibility to produce different parts in different ways, process flexibility to maximise parts types without major setup, product flexibility to create new parts, routing flexibility to produce parts through alternate routes through the production process, volume flexibility to adjust output levels, expansion flexibility to adjust capacity and capability as needed, program flexibility to run autonomously for long periods, production flexibility to manufacture a multitude of parts without new capital equipment, and market flexibility to adapt to market changes [32]. Maximum flexibility implies the ability for rearrangement, change in materials and machining, machining more complex geometries to increase the product range and variation, and the ability to integrate new machining technologies. Recently, lean production has emphasised a reduction in inventory stock (production to demand rather than stock for supply), rational sequencing of operations and the elimination of waste—indeed, the lunar industrial ecology implements this through recycling. Lean manufacturing combines the high-quality customisation of craft production with the high quantity cheapness of mass production, a task to which 3D printing is eminently suited. Examples of this include JIT manufacturing (minimise excess inventory by matching production rate to demand) and total quality management TQM (minimise product waste through continuous quality control). JIT manufacturing bears similarities to generalised assembly line balancing along a conveyor to consecutively distribute the total manufacturing workload along the flow line. A variation of TQM is 6 sigma quality which aims to reduce tolerance deviance beyond the traditional 3 sigma levels at every stage of the production process to minimise defective production. All are effectively concerned with the minimisation of waste.

## 5. Manufacturing Architectures

Centralised architectures offer complete global control effort but are slow to respond to perturbations due to high overheads. This may be modified into a top-down hierarchy which overcomes the overhead problem through task decomposition but the response problem remains. The hierarchy is the most traditional organisational form with its tree-like structure where fewer higher agents have more global views than the more numerous lower agents in the hierarchy. Complex systems often form hierarchies of interrelated subsystems in which the interactions between subsystems are suppressed with respect to those within subsystems—in this case, they are nearly decomposable such that subsystems can be treated as if they are almost independent of each other. This is the principle of decomposition of increasing precision with decreasing intelligence [33]. An example applied to FMS is a three-layer hierarchy of an organisation level for scheduling integrated sets of machines (factory level), coordination level for coordinating machines (cell or job shop level) and execution level for controlling each machine task (machine level) [34]. Hierarchies implement a divide-and-conquer strategy but are brittle. The hierarchy may or may not have same-level interactions through internal links. Control architectures in manufacturing have evolved from centralised hierarchies into distributed heterarchies [35]. Hierarchies may be modified by allowing some same-level interactions in a more distributed approach. The heterarchical architecture is a flexible distributed multi-agent approach to problem solving in which no single agent has a global view of the problem, only a partial view. Blackboards such as the NASREM control architecture are of this nature [36]. The true heterarchical structure is flat characterised by entirely same-level interactions in a distributed fashion without any central or global control. In biological systems, distributed control of modules operating using only local information without centralised arbitration is ubiquitous. As long as communication exists between these subunits, coordination through a higher level self-organised centralised controller is possible [37]. The distributed heterarchy is highly fault tolerant to perturbations but is difficult to predict. Decentralised approaches may be combined with supervisory control to form hybrid hierarchical/heterarchical information structures. 

Complex multi-agent manufacturing systems may be configured in several different ways. Agent-based systems are ideally suited to distributed architectures. Agents are objects of the object-oriented paradigm in which a set of tasks interact through message passing. However, whereas object-oriented approaches are built around passive objects, agent-based approaches place emphasis on active agent interactions. Agents involve autonomous well-defined entities with well-defined boundaries and interfaces [38]. They are cooperating autonomous entities that can self-organise into a population without any global controller—each agent has knowledge for its own task. Each agent has an interface to interact with other agents and/or the environment and each agent is hierarchically constructed. There are many applications of agent-based computing [39]. CORBA (common object request broker architecture) is an industrial middleware agent-based protocol applied to intelligent machine cells to coordinate their subsystems [40]. An open systems architecture can partition the FMS into autonomous entities (modules) that communicate and coordinate with each other [41]. Agents must learn from their environments to adapt—with multiple agents, this is a complex task requiring mechanisms for coordination. There are several market-based approaches to multi-agent mechanisms [42]. The bucket brigade algorithm optimises work allocation between resources to maintain load balance without supervision. The contract net protocol is a market-based technique that involves agents competing for subtasks through the submission of bids [43]. However, optimisation-based allocation of tasks among agents is superior to market-based approaches in multi-robot task allocation [44,45]. Optimisation generally implies linear programming with respect to utility, fitness value or resource cost of a task [46,47]. The evolutionary algorithm is an optimisation procedure that is suited to manufacturing schedules [48]. Similarly, it has been proposed that an information-theoretic measure—generalised correlation entropy—of spatiotemporal coordination of multiple modules of a distributed robotic system be employed as the fitness function of a genetic algorithm to evolve the system [49]. 

Self-organisation can be applied to multi-agent manufacturing in which multiple agents form a society of agents to solve problems beyond any individual agent’s capacity [48]. Agents have only local interactions and interact with each other through a coordination model. Local interactions between components result in emergent global properties without any central control or supervision. For self-organisation, a critical threshold must be exceeded for the emergence of global order to occur. It is the multiplicity of short-range interactions that yield complex “emergent” global behaviours that are not reducible to the behaviour of its parts. The commonest example is the collective behaviours of insect colonies of ants, termites, bees and wasps. The ant colony is a self-organising system in which stigmergy provides the mechanism for implicit rather than explicit coordination [50]. Each ant observes cues from its environment independently to invoke simple behaviours individually. By depositing volatile chemicals, pheromones, some fluctuations grow while others fade over time. These accumulating pheromones drive individual agents to appear coordinated. Communication between insects is indirect and mediated through the environment—stigmergy. Stigmergy implements communication between components through modification of the local environment. Individual insects mark their environment with pheromones which collectively coordinate them. Ants modify their environment by depositing pheromones locally and the pheromones propagate spatially as a global dissipation field. It is these local modifications that communicate to other foraging ants. A specific sign in the environment triggers specific actions by the agents. This can be directly applicable to robot agents in a mining, chemical processing and manufacturing environment. Swarms of multiple agents with simple local behaviours can generate complex behaviours with multi-robot coordination. 

Fully autonomous mining, chemical processing and manufacturing will require self-learning and self-optimisation to adapt flexibly and rapidly to a variable demand environment, i.e., reconfigurability is essential [51]. There are certain design principles required for reconfigurability that goes beyond traditional FMS [52]. They are based on flexibility, convertibility, scalability and modularity and the key element is the implementation of actuation to provide multiple degrees of freedom. Reconfigurability reduces the complexity and cost of FMS by adaptively matching capacity to need [53,54]. Reconfigurable manufacturing systems are designed to accommodate rapid changes in the manufacturing architecture in response to new demands of production. Reconfigurability minimises unused capacity but adjusts rapidly to new demands [55]. This rapid response to changing demands is the hallmark of agile manufacturing (itself similar to lean manufacturing except that agile manufacturing is proactive while lean manufacturing is reactive) [56,57]. Reconfigurable systems are distinct from FMS in that they are rapid but they are limited in part diversity whereas FMS has maximum flexibility in part range. We need both. There are six principles of reconfigurability [58,59]: (i) modularity; (ii) integrability of interfaces; (iii) customised flexibility; (iv) scalability of factory; (v) convertibility of factory to different production requirements; (vi) diagnosability of abnormal behaviour. Modules can be reconfigured rapidly into an integrated system which can be readily modified with new modules to adjust both product and capacity. Modular design of products permits agile manufacturing by configuring modules into different products, the configurations of which can be searched using tabu search [60]. Tabu search is suitable for solving NP-hard problems by starting from an initially feasible solution to search for better solutions subject to minimum cost constraints. Genetic programming also may be employed to evolve the self-organisation of parts into a final self-assembly [61]. The genetic program has a hierarchical structure with components for assembly that can be randomly selected, subject to assembly constraints defined by a fitness function. Reconfigurable manufacturing allows ready flexibility to perturbations that would affect throughput without re-designing the manufacturing plant. Many reconfigurable systems employ material transport systems such as gantries and conveyors that form the backbone of the system with an emphasis on CNC machining. The flows of service or goods in such a transport network are controlled by demand and supply in a market economy system forming a network topology reminiscent of a genetic regulatory network. The volume of service or goods through the transport network may be quantified by Kirchoff’s circuit laws. Petri nets may also be used to model concurrent manufacturing activities. There are several types of reconfigurable manufacturing architecture affording dynamic flexibility of distributed cells—fractal, holonic and bionic [62]. The units in each case are slightly different. The fractal manufacturing architecture is a reconfigurable system whose chief characteristic is that its autonomous agents are self-similar and reconfigurable and these agents cooperate through message passing to solve problems [63,64]. They are self-similar at all levels of their hierarchy, the configuration of which is controlled by a system agent. It can autonomously self-organise its organisational structure (but not its physical structure) in response to a dynamic environment through reinforcement learning [65]. Reinforcement learning lies between the exact feedback of supervised learning and the lack of feedback of unsupervised learning. It is unclear how the fractal architecture might be implemented practically.

## 6. Bioinspired Manufacturing Architectures

**Figure 2:**
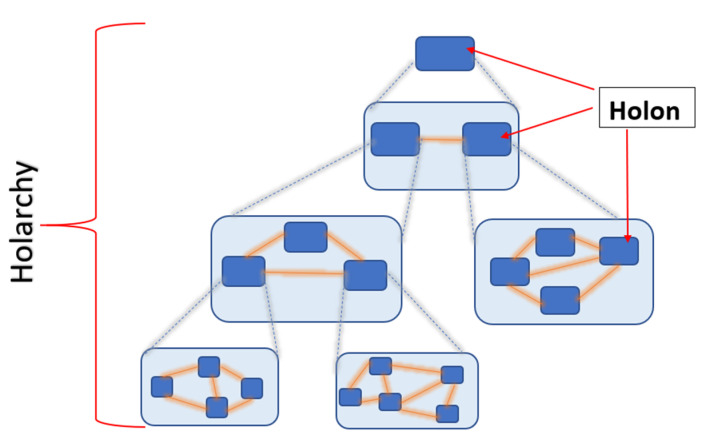
Holonic architecture of holons.

The holonic architecture is a bio-inspired approach that implements the reconfigurable factory of the future (Figure 2). The holonic architecture is the most popular agent-based approach to manufacturing systems control as it combines hierarchical and heterarchical architectures [66]. Life is a complex system in that it is characterised by multiply interacting agents which themselves may be simple with nonlinear interactions subject to simple short-range laws. Holony is based on Arthur Koestler’s concepts on the material basis of mind-brain duality in his “Ghost in the Machine” (1967) [67]. Biological cells are comprised of organelles while also being part of tissues. Inspired by organelles of the biological cell, the holonic factory model comprises a hierarchy of tissue holons comprised of multiple cell holons. Each cell holon comprises a nucleus holon (for decision-making), a golgi complex holon (for inventory), a lysosome holon (for reprocessing and recycling), an endoplasmic reticulum holon (for transport) and ribosome holons (for production) in which mRNA act as a messaging system and tRNA as a negotiation system. The holarchy is a system of holons that permits a heterarchical system to implement a nested hierarchical structure to provide conflict resolution with the holon representing a hybrid character of both whole and part [68]. A holon is simultaneously both a subordinate agent comprised of parts from a lower level and part of a larger superordinate agent. The holonic architecture self-regulates in response to perturbations from the environment modelled as a social system [69]. It is based on cooperating holons forming an integrative holarchy based on functional decomposition. The holarchy combines the static stability of the hierarchy with the dynamic flexibility of the heterarchy through the dual nature of the holon [70]. The holon is an autonomous, self-contained, self-regulating module yet it is part of higher order holons and itself is comprised of lower order holons, smearing the difference between part and whole, i.e., holons may be aggregated (exhibiting emergent complex behaviours from interactive simple behaviours) or specialised (exhibiting the inheritance of agents). The holarchy comprises different sets of alliances—short-lived coalitions, task-oriented teams, long-lived congregations, long-lived societies governed by social laws, loosely bound federations, etc. Holons interact through broadcast messaging and the contract net protocol. Metamorphic control of holonic systems is an approach for real time operation [71]. The holarchy can be augmented with stigmergy to enhance clustering through self-organisation [72]. An example of an holonic system is the plug-and-produce software reconfiguration facility of an holonic robot assembly system comprising three manipulators, one belt conveyor and two warehouses [73,74]. The holonic system has been adopted in automotive factories.

Product–resource–order–staff architecture (PROSA) is an agent-based holonic manufacturing reference architecture based on four types of holonic agents [75,76]: (i) order holons (agents for workpiece tasking, logistics and its control and timing); (ii) product holons (agents for product functionality such as process planning and quality assurance); (iii) resource holons (agents for physical and information resource handling such as machine or factory); and (iv) staff holons (agents for global centralised supervision). Software agents acting as virtual ants coordinate between physical agents so they can aggregate multiple agents to form holonic systems at multiple levels. PROSA has a self-similarity aspect incorporating a fractal architecture. Stigmergy has been demonstrated within the context of PROSA as an indirect mechanism of coordination in a multi-agent holonic manufacturing system [77,78] in which PROSA agents were coordinated for adaptability to changes in the environment. In this case, termite-inspired robots using only very simple behavioural rules could build structures from magnetically-connected bricks through emergence. PROSA may be extended ontologically into the bionic architecture based on a hierarchy of biological cell analogues (modelons) including the possibility of biological morphogenesis. A cell may be differentiated into different functions but all are based on the same underlying architecture.

The Biological Manufacturing Systems is a bio-inspired approach to manufacturing for coping with internal and external environmental perturbations during the product lifecycle. In this paradigm, manufacturing machines breed products in which potential fields attract dynamically jobs to machines [79]. A classifier system with if-then production rules with bucket brigade credit assignment of weightings was adopted to implement genetic learning [80]. A neuroendocrine-inspired manufacturing system (NEIMS) emulates the biological neurocontrol-hormonal regulation system and its characteristic adaptability [81]. The endocrine system releases hormone signalling molecules through the hypothalamus-pituitary-adrenal axis to the bloodstream in response to neural stimulus—hypothalamic neurons stimulate pituitary CRH (ACTH-releasing hormone) to stimulate adrenal ACTH (adrenocorticotropic hormone) synthesis which in turn stimulates biosynthesis of cortisol which inhibits CRH/ACTH production. The nervous system implements adaptive control while the endocrine system implements biochemical homeostasis. NEIMS implements hierarchical neural control under nominal conditions but switches to hormone regulation for agile adaptation under off-nominal conditions with a reorganisation of resources. Hormonal secretion quantity is given by [82] $\rho_{ij}=\frac{a}{c_{ij}}{\left(1+\lambda \right)}^{t-1}$ where *a* = constant, *c_ij_* = position-dependent cost for task, *t* = duration of job, and *λ* = control parameter. This latter is particularly analogous to the lunar industrial ecology of chemical processors. Indeed, it is modelled on a specific example of a metabolic system. 

## 7. Metabolic Network Modelling

**Figure 3:**
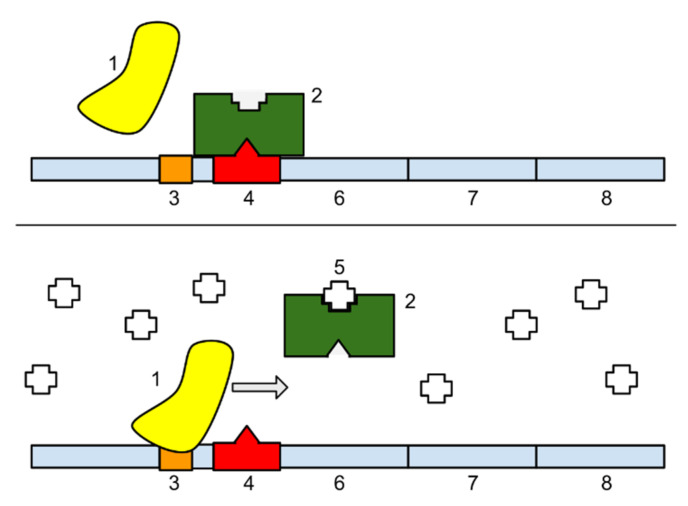
Lac operon (top) repressed; (bottom) active: 1 = RNA polymerase; 2 = repressor; 3 = promoter; 4 = operator; 5 = Lactose; 6 = lacZ; 7 = lacY; 8 = lacA (Creative Commons Attribution—Share Alike 3.0 credit T A Raju).

Metabolism constitutes a set of material causal interrelations associated with thermodynamic energy flows in biological cells and organisms. There are several network representations of the biological cell [83]: (i) metabolic networks represent chemical reactions between metabolites; (ii) protein networks represent protein-protein interactions including signalling networks; (iii) gene networks represent relationships between genes through genetic expression. The relationship between these networks is complex in the genetic expression is mediated through RNA processing. The Monod-Jacob operon model of gene regulation is based on two operations—inducer and repressor. The lac operon of *Escherichia coli* requires an inducer (allolactose) to determine expression of the lac operon [84] (Figure 3). The inducer binds to the lac repressor which prevents its binding to the lac operon so LacZ protein is produced. Without the inducer, the lac repressor binds to the lac operon so LacZ protein is repressed. 

This is a bistable system based on a threshold level of the inducer (allolactose) with the net rate of inducer concentration determined by the difference between inducer production rate *v_in_* and inducer removal rate *v_out_* [85]:

$$\frac{d\left[a\right]}{dt}=v_{in}-v_{out}$$

Removal of the inducer occurs through binding to the repressor and catabolism such that:

$$v_{out}=v_{cat}-v_{bind}=k_{cat}\left[a\right]+k_{bind}\left[a\right]=k\left[a\right]$$

The rate of inducer production has a sigmoidal relation to inducer concentration given by the Hill extension of Michaelis–Menten kinetics:

$$v_{in}={\left[a\right]}_{max}\left(k_{0}+\frac{{\left[a\right]}^{n}}{K+{\left[a\right]}^{n}}\right)$$

where *K* and *n* are sigmoidal shape parameters, *K* = Michaelis–Menton constant, *n* = Hill coefficient of molecular binding, *k*_0_ = basal diffusion of inducer. The sigmoid generates the two states of the lac operon depending on [*a*]: low [*a*] represses the operon while high [*a*] expresses the operon. The lac operon may be described by a Boolean function (Table 2) [86].

If *k*_0_ = 0 and *n* = 1, we have the Michaelis–Menten equation used for modelling metabolic reactions [87]. Gillespie’s algorithm is a more sophisticated stochastic simulator of biochemical networks within the biological cell modelling a well-mixed bioreactor in which chemical reactions are stochastic [88].

The lac operon implements a logical implication modelling an “if…then…” function that provides the basis for production rule based expert systems. It may be considered that an expert system will be necessary to enable autonomous operation of the chemical processes of the ISRU unit. This will be particularly important for continuous operations and which supply mission-critical resources such as oxygen for life support and fuel. Chemical processing is not a deterministic process due to random or unknown variations. The expert system is used primarily to diagnose faults indicating off-nominal performance to ensure sufficient production. In turn, this implies that the production process must be simple and robust to minimise human interaction. It has been suggested that rule-based diagnostic expert systems using a dynamic chemical model would be suitable for ISRU systems [89]. The simplest model assumes a continuous supply of reagents with input flow mass controlled and monitored output mass of both products and waste. Intermediate mass flows must also be monitored and regulated through operating conditions of flow rate, pressure, temperature, voltage and current—temperature in particular is a sensitive control variable. For gases such as in a Sabatier reactor, this is relatively simple. Petri nets offer effective process models of throughput and dependencies. However, the most difficult faults to compensate for are those involving mechanical failures that yield ruptures to throughput, i.e., leakages or blockages in mass flow lines. There may be electronic faults due to excess voltages from the power supply, thermal inertia delays yielding higher than desired temperatures, etc. The most drastic solution is immediate shutdown. This is where a scalable cellular approach is advantageous in that it yields graceful degradation allowing continued operations in the event of individual cell failures. There must be extensive facilities for self-health checkout, fault detection and isolation, fault correction through simulation models and emergency shutdown procedures. Fault detection, isolation and correction require the use of adequate process models. These must be augmented by sensor failure models to incorporate inaccurate information. The most appropriate approach to this type of diagnostic modeling is fuzzy control methods [90]. Model predictive control provide the basis for self-diagnosis of faults. An estimate of different dynamic models is generated from different plant input/output data. The current input/output datasets determine the model which predicts the plant’s future response up to a future horizon. Alternatively, qualitative simulation is based on semi-quantitative models of state transitions which express imprecision through numeric ranges [91]. 

## 8. Genetic Regulatory Networks 

**Figure 4:**
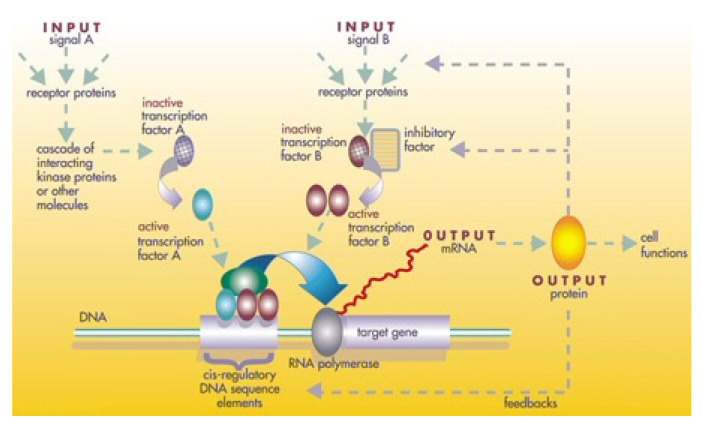
Genetic regulatory network (credit US Department of Energy).

Much of gene regulation occurs at the level of transcription into mRNA by ensuring the energy is not wasted on unnecessary activities. Bacterial genes are organised as operons that include regulatory DNA which control the transcription of the operon. Regulatory regions to which regulatory proteins bind promote or inhibit transcription downstream. Regulatory proteins are usually also activated or deactivated by inducers or repressors by affecting their binding affinity to DNA through shape changes by enhancing or blocking RNA polymerase activity at the promoter site. The genes for each of these proteins are clustered into a single operon. The genetic regulatory network (GRN) is a mechanism through which prokaryotic and eukaryotic organisms use a network of regulators to interact and control gene expression to manufacture mRNA/proteins in response to a changing environment from multiple environmental signals. We shall review GRNs and their modelling as networks (Figure 4). Gene expression is mediated primarily through the interaction of transcription factors (regulatory proteins) with specific DNA sequences in the control region of the genome located separately from the protein coding region that it controls. Binding of transcription factors to the regulatory region (promoter) acts as a molecular switch that activates RNA polymerase. In prokaryotes, only one or two transcription factors are involved but eukaryote gene expression is more complex with several transcription factors forming a transcription complex. The problem with single transcription factors for single genes is that it imposes an infinite regress problem. Employing different combinations of transcription factors for different genes, a small number of regulatory proteins can control a large number of genes. Each gene is switched on unless it is switched off by a specific repressor that blocks the binding of RNA polymerase. In the absence of the repressor, the gene is expressed only at a basal level. Higher gene expression levels require an activator. In higher eukaryotes, enhancers bind to activators to further stimulate transcription. Genetic networks involve highly connected protein signal pathways with feedback between many genes that control cellular operations. GRN are the basis of biological cell function in controlling growth, differentiation into cell types and response to the environment. They are the networks of mutually activating and repressing genes and their gene products forming complex circuits. Binding of repressor proteins to specific DNA motifs prevents RNA polymerase from transcribing specific downstream genes. Regulatory genes control transcription factors through activation or repression of specific control regions forming a web of transcription factors for gene expression [92]. 

A superior early review of DNA binding motifs is given in [93]. The DNA double helix yields a major groove and a minor groove whose width and depth are determined by the specific DNA sequences. The binding sites of proteins are complementary with a protruding shape (typically an α-helix) that fits into the major or minor grooves of DNA. High specificity of the binding for gene expression requires a significant DNA binding length. There are a number of different motifs that serve DNA recognition. The basic leucine zipper involves a number of transcription factors with repeating Leu residues spaced seven residues apart forming an α-helix coiled coil. The TATA box binding protein initiates transcription of all three polymerases in eukaryotes. Two β loops form a pair of stirrups that wrap around the DNA grooves by deforming the TATA sequence to initiate unwinding of DNA. The zinc finger motif is a repeated sequence of a 25–30 amino acid sequence module with two His, two Cys and three hydrophobic amino acids. Zinc fingers are the specific component of transcription factors with zinc ions in combination with cysteine and histidine residues (e.g., Cys_2_His_2_ fold) that define their DNA sequence binding affinity to the GC box. Each zinc finger module forms a folded zinc-containing domain, a multitude of which forms a sequence-specific motif. Zinc fingers are the most widely adopted DNA binding motifs including two-zinc fingers and three zinc fingers being common. With the three zinc fingers motif, specific recognition is achieved by each single amino acid-base pair interaction forming a triplet of base pairs. Hormone receptor DNA binding domains are a class of two zinc fingers in which each finger comprises just two pairs of Cys rather than a pair of both Cyc and His. There are other classes of zinc finger DNA-binding domains in which Zn^2+^ is employed to fold proteins into compact domains required for binding to nucleic acids. 

Transcription factors are regulatory proteins that bind to DNA motifs signifying regulatory genes and are often activated through phosphorylation. Phosphorylation, acetylation, methylation and ubiquitination are protein modification mechanisms associated with the regulation of gene expression. Phosphorylation is the process of adding a phosphate group to a protein catalysed by kinase enzymes and reversed by phosphatases. The most basic protein dynamics is characterised as a balance subject to the law of mass action (e.g., between anabolism/catabolism or phosphorylation/dephosphorylation) [94]:

$$\frac{dR}{dt}=k_{0}+k_{1}S-k_{2}R$$

$$\frac{dR}{dt}=k_{1}S\left(R_{T}-R_{P}\right)-k_{2}R_{P}$$

where *S* = [mRNA] = signal, *R* = [P] = unphosphorylated protein response, *R_P_* = [*R_P_*] = phosphorylated response, and *R_T_* = [*R_T_*] = [*R* + *R_P_*] = total response. One steady-state solution is given by:

$$R=\frac{k_{0}+k_{1}S}{k_{2}}\;\mathrm{and}\;R_{P}=\frac{R_{T}S}{(k_{2}/k_{2})+S}$$

If phosphorylation/dephosphorylation reactions are mediated by Michaelis–Menten reaction kinetics:

$$\frac{dR_{P}}{dt}=\frac{k_{1}S\left(R_{T}-R_{P}\right)}{k_{m1}+R_{T}-R_{P}}-\frac{k_{2}R_{P}}{k_{m2}+R_{P}}$$

The steady-state concentration of phosphorylated protein is given by the solution to the quadratic equation:

$$k_{1}S\left(R_{T}-R_{P}\right)\left(k_{m2}+R_{P}\right)=k_{2}R_{P}\left(k_{m1}+R_{T}-R_{P}\right)$$

The shape of a genetic regulatory function determines its temporal response to input (environmental) signals, e.g., feedforward loop in which two genes X and Y both regulate a common gene Z which is insensitive to minor fluctuations, i.e., noise [95]. There are often multiple phosphorylation sites on a protein. In the embryo of *Xenopus*, MAPK (mos-nitrogen-activated protein kinase) is a family of kinases that phosphorylate a set of proteins activated by MAPK kinase that itself is activated by MAPKK kinase—this MAPK cascade is activated by the steroid progesterone. In response to extracellular signals, cyclic AMP activates the phosphorylating enzyme protein kinase A that regulates genetic transcription factors such as cAMP response element binding protein (CREB) that binds to the nucleotide sequence TGACGTCA [96]. The motifs—cis-regulatory control modules—form the core of genetic regulation and these modules are re-used by different genes [97]. Cis-regulatory control modules are wired together to form complex network circuits implementing combinatorial logic functions [98]. Diffusion of transcription factors implements information transmission with only local synchroneity. GRN determine which genes are transcribed and expressed. Positive and negative feedback loops between genes implement regulation of transcription with circular chains of interaction. When bacteriophage λ infects a bacterium and lyses the host cell (host cell destruction—Xis), it releases a short arbitrium peptide—the greater the number of bacterial cells infected, the greater the peptide signal until the phage implements a state of lysogeny (prophage integrated into host DNA—Int) due to quorum sensing, i.e., viruses communicate [99]. A genetic circuit decides between lysogeny and lysis depending on feedback on the state of the environment. GRN logic may be simulated through electrical circuit representations in which biochemical repression or activation of transcription act as active logic gate functions with signal time delays to simulate transcription and signal accumulation times [100]. Cl and Cro regulatory proteins control promoter repression at O_R_1 and O_R_3 sites repressing the synthesis of each other with complex logical rules:

“Int is produced” IF “CI is above threshold” OR “(“state is prophage” AND “P_L_-initiated RNAP is present”)

“Xis is produced” IF “P_L_-initiated RNAP is present” NAND “CII is present”

A switch selects between two stable configurations determined by Cl or Cro negative feedback loops that selects between lysis and host integration:

“Next state is prophase” IF (“current state is prophage” NAND “Int above threshold”) OR (“Int above threshold” NAND “Xis above threshold”) 

This bacteriophage λ gene regulatory circuit is very robust to genetic changes to individual components of the control system [101]. Genetic regulatory circuits are typically modular as their genes are co-located but their wiring into more complex functions may be subject to considerable evolutionary plasticity as an adaptation to environmental complexity [102]. Transcription network motifs are highly conserved sets of recurring regulation patterns forming the building block of GRN [103]. A significant fraction of motifs involve negative autoregulation in which a transcription factor represses the transcription of its own gene, e.g., threonyl tRNA synthetase represses the transcription of its own mRNA. This motif accelerates up response time when production is below threshold beyond which it slows. Positive autoregulation involves a transcription factor that enhances its own rate of production—it slows response time when production is below threshold. Autoregulation through negative feedback is crucial to gene network stability [104]. The feedforward loop (FFL) is a three-gene network motif comprising two input transcription factors X and Y, one regulating the other and both of which regulate a target gene Z [105]. X and Y may be configured as an AND gate or an OR gate. Of eight possible activator/repressor interactions, four act as signed accelerators that speed up response time of gene expression to stimulus in one direction only while the other four act as signed delay elements, e.g., flagella gene expression is prolonged after input signal is stopped but no delay occurs with active inputs. FFL can be combined into more complex transcription circuits—interlocking FFLs control differentiation of *Bacillus subtilis* into spores under starvation conditions by switching genes ON and OFF through a series of temporal waves to generate sequences of sporulation stages that exploit FFL acceleration and delays.

Biological GRNs are complex and require modelling as networks. GRN modelled as a graph G with nodes representing genes with edges representing causal relations between genes, i.e., activation/repression of transcription factors. Co-expressed genes with similar expression activity are assumed to be related in function through correlation measures (such as Pearson’s correlation coefficient). However, gene duplication can alter patterns of activity in genetic networks. There are four main methods for modelling genetic networks—random Boolean networks, differential equations, relevance networks and Bayesian networks [106,107,108]. Random Boolean networks are abstract representations of genetic and metabolic networks that can replicate spontaneous self-organisation. In random Boolean networks, networks of N genes are represented as randomly interconnected sets of binary switches (gene on/off) [109]. Each node’s state is determined by a Boolean logic operation on its inputs. The initial node state is random but it evolves depending on their interconnections. If each gene is randomly linked directly to K other genes, there are 2^2K^ possible Boolean functions. Each gene is controlled by a randomly assigned Boolean function from the ensemble of all possible NK Boolean networks. A sequence of states forms a state trajectory which is characterised by different attractors—point attractor, limit cycle or strange attractor. Such networks may exhibit edge-of-chaos dynamics, a phase transition between order and chaos [110]. When *K* = 2, the network dynamics exhibits short stable behaviour cycles at the edge of chaos. It predicts cell replication time and the number of cell types ($\sqrt{N}$) as the number of attractors in terms of gene number. If *K* = 1, the network is highly ordered; if *K* = 3, the network becomes chaotic. The *K* = 2 condition generates cyclical dynamics implying that complex GRN may be decomposed into simpler weakly interconnected regulatory modules with localised feedback loops [111]. GRNs must be both robust to genetic errors—point mutations, gene duplications and gene deletions—and adaptable (evolvable) into new states [112]. Gene duplications with divergence in particular offer the prospect for high evolvability coexisting with genetic stability. This requires edge-of-chaos dynamics between order and chaos (instability). The Boolean network exhibits the robustness of GRN afforded by the connectivity *K* ≥ 2 [113]. It has been suggested that random Boolean networks modelling activation patterns of GRN can implement low-density parity check codes [114]. 

Although Boolean networks can exhibit complex logical relationships represented by wiring diagrams, they do not model real biological systems well. Metabolic networks across all three domains of life on Earth are topologically similar as scale-free networks in which the probability of k links between nodes is given by a power law $P\left(k\right)\;\approx \;k^{-\gamma }$ where γ = 2.2–2.5 [115,116]. Their topology is dominated by a few highly connected hubs in that a few hubs are highly connected but most nodes are relatively localised. Most genes are regulated by only one to three transcription factors but a few transcription factors are common to many genes. In metabolism, only three or four metabolic reactions link most pairs of metabolites—these are commonly remnants of the RNA world including coenzyme A, NAD and GTP that form the core of glycolysis and the tricarboxylic acid (Krebs) cycle [117]. This is the bowtie architecture in which highly conserved core processes such as the Krebs cycles are highly conserved but funnelling many inputs and many outputs. The bowtie is robust to evolutionary perturbations by permitting a multitude of inputs to the core process. Our lunar industrial ecology exhibits a bowtie architecture (essentially a de Laval nozzle architecture), a strategy common in complex biochemical pathways to control the complexity of biochemical networks. A large suite of input streams fan-in to a small set of processes (the throat) to generate a large fan-out of products. In our case, our throat comprises the Metalysis FFC process and a small suite of additive manufacturing processes. High clustering of highly interconnected modules form functional modules that the scale free architecture combine into hierarchical modules. Modularity at hierarchical scales is ubiquitous in biological systems—the biological cell is a module of multicellular organisms, itself comprised of organelles downscale and forming the basis of tissues and organs upscale. Hierarchical modularity is the essence of the holonic architecture. All major cellular functions are executed by modular networks, e.g., Krebs cycle [118]. The widespread incidence of such modularity in biological networks is a direct result of evolutionary selection to reduce the costs of connections to ensure evolvability [119]. The small world topology means that any two nodes can be connected with a path of just a few links. Scale-free networks are robust to random errors. The scale-free topology results from evolutionary expansion with the addition of new vertices which preferentially attached to highly connected nodes through gene duplication [120]. It is apparent therefore that gene networks are not randomly connected as represented by random Boolean networks. 

Ordinary differential equations may be employed to model chemical reaction kinetics of GRN constrained by balancing mass action between two species concentrations:

$$\frac{dX}{dt}=v_{syn}^{X}\left(Y\right)-v_{deg}^{X}\left(X\right)$$

$$\frac{dY}{dt}=v_{syn}^{Y}\left(X\right)-v_{deg}^{Y}\left(Y\right)$$

Genetic expression of genes is given by the weighted sum of all genetic expression at the previous time step:

$$\frac{\delta x_{i}\left(t\right)}{\delta t}=f\left(\sum_{j=1}^{n}w_{ij}x_{j}\left(t-1\right)+b_{i}\right)$$

The weight matrices *W*, where *w_ij_* represents the regulatory influence of gene *i* and gene *j* and the entire matrix models interactions between all *n* gene combinations. The simplest model is that of cross-regulation of two genes through activator/repressor binding [121]: (i) if both proteins are either activators or repressors, a single bifurcation yields bistability for *n* ≥ 2, i.e., genetic switches; (ii) for combinations of activators and repressors, Hopf bifurcations result in undamped oscillations for *n* > 2. In the two-gene network of gene expression, the ratio of noise variance to the noise mean (Fano factor) as a measure of noise amplitude for both proteins must exceed unity [122]. GRN and PPI (protein-protein interaction) networks may be integrated [123]. In general, GRN in discrete recursive form is given by mRNA expression level of gene *i*:

$$x_{i}\left(t+1\right)=x_{i}\left(t\right)+\sum_{j=1}^{n}a_{ij}z_{j}-\alpha_{i}x_{i}\left(t\right)+k_{i}+n_{i}\left(t\right)$$

where *x_i_*(*t*) = mRNA expression level of gene *i* at time *t*, $z_{j}\left(t\right)=f_{j}\left(y_{j}\left(t\right)\right)=\frac{1}{1+\exp\left(-\frac{y_{j}\left(t\right)-u_{j}}{\sigma_{j}}\right)}$=*j*^th^ transcription factor protein binding to gene *i* modelled as a sigmoid function, *a_ij_* = regulatory ability of *j*^th^ regulatory gene to the *i*^th^ expressed gene, *α_i_* = degradation factor from time t to *t* + 1, *k_i_* = basal level, and *n_i_*(*t*) = stochastic noise. PPI in discrete recursive form is given by protein activity level of gene *i*:

(1) $$y_{i}\left(t+1\right)=y_{i}\left(t\right)+\sum_{j=1}^{m}b_{ij}y_{i}\left(t\right)y_{j}\left(t\right)-\beta_{i}y_{i}\left(t\right)+\gamma_{i}x_{i}\left(t\right)+h_{i}+v_{i}\left(t\right)$$

where *y_i_*(*t*) = protein activity level of gene *i* at time *t*, *y_j_* = activity of *j*^th^ protein interacting with protein *i*, *b_ij_* = interaction ability of *j*^th^ interactive protein with the *i*^th^ protein, *β_i_* = degradation factor from time *t* to *t* + 1, *γ_i_* = translation effect from mRNA *x_i_*(*t*) to protein *y_i_*(*t*), *h_i_* = basal level, and *v_i_*(*t*) = stochastic noise. The transcription factors and translation parameter *γ_i_* of gene expression of protein act as the coupling mechanisms between GRN and PPI.

Relevance networks define the interaction between two genes *i* and *j* through mutual information I for all gene pairs:

$$I\left(x_{i},x_{j}\right)=\sum_{x_{i}}\sum_{x_{j}}p\left(x_{i},x_{j}\right)log\left(\frac{p\left(x_{i},x_{j}\right)}{p\left(x_{i}\right)p\left(x_{j}\right)}\right)$$

where $p\left(x_{i},x_{j}\right)$ = joint probability distribution of *x_i_* and *x_j_*, and $p\left(x_{i}\right)$, $p\left(x_{j}\right)$ = marginal probabilities. The chief problem with relevance networks is that they are bidirectional. Bayesian belief networks represent interactions between genes as joint probabilities of conditional dependence, $p\left(x\right)=\overset{n}{\underset{i=1}{\prod }}p(x_{i}|pa(x_{i}))$ where *pa*(*x_i_*) = parent of *x_i_*. Learning a Bayesian network is a NP-hard problem and Bayesian networks cannot represent circular dependencies of gene relations. Dynamic Bayesian networks resolve the latter by mapping them onto sequences of acyclic events. Approximation techniques such Markov Chain Monte Carlo resolve the former. Bayesian networks then represent an appropriate representation of GRN in incorporating logic, probability and network properties that could potentially be applied to our lunar industrial ecology. 

## 9. Biological Cell Cycle

**Figure 5:**
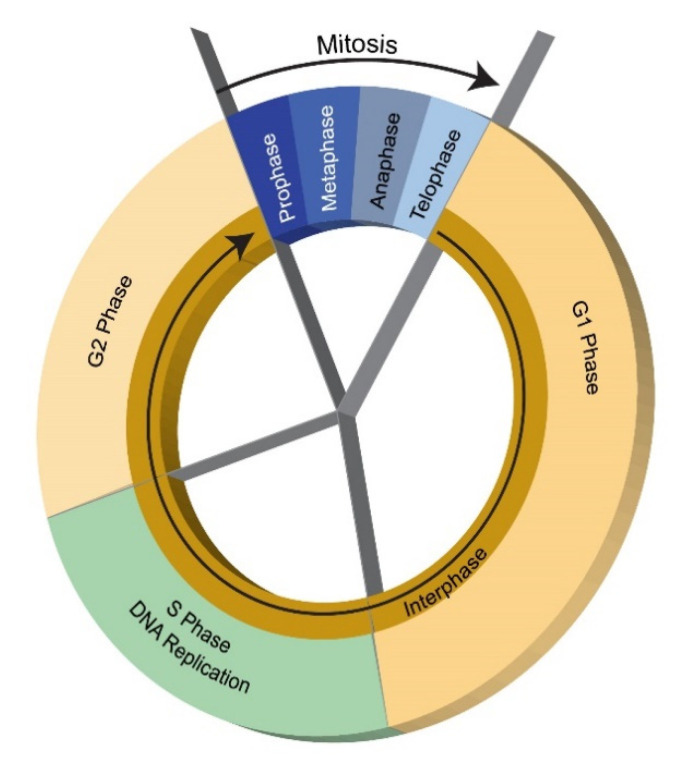
Cell cycle (credit National Institute of Health).

The bacterial cell cycle is under closed loop control of a small number of discrete regulatory proteins organised as a cyclical genetic circuit [93]. Feedback and feedforward signals in GRN generate sigmoidal switches (buzzers), transient responses (sniffers), hysteretic switches (toggles) and oscillators (blinkers) as components for complex regulatory and signalling circuits [124]. The animal cell cycle of replication follows through four phases every 10–30 h in tissue culture (except when in a non-growing quiescent G0 state)—G1 (unreplicated chromosomes)—S (DNA synthesis)—G2 (replicated chromosome)—M (mitosis) [125] (Figure 5). Collectively, G1-S-G2 form the interphase between mitosis events—the mitosis phase itself is subdivided into four subphases—prophase (DNA condenses into chromosomes and nuclear membrane dissolves releasing its contents into the cytoplasm), metaphase (chromosomes attach to mitotic assembly at each opposing side of the cell), anaphase (chromosomes are pulled apart into opposing sides of the cell) and telophase (two nuclear membranes reform, cell contents redistribute and two cells separate). Different cell types have different cell cycle durations due to variations in G1 with the other phases being relatively constant—S (6–8 h)—G2 (2–6 h)—M (1 h). DNA is replicated in the S state and the two chromatids are segregated in the M state. Transition to G1 state with DNA synthesis or G0 quiescent state is determined early in the cell cycle by biochemical signals. If in the G1 state, rRNA and tRNA proliferate prior to entering S state; G0 state is entered if G1 events cannot be completed. Chromosomes must be aligned and attached to the spindle in metaphase. MtDNA replication is independent of and occurs continuously throughout the eukaryotic cell cycle. Cells use checkpoint controls that prevent initiation of a sequence of events by maintaining a checkpoint state until a feedback signal is transmitted on completion of an earlier sequence. Checkpoints involve a monitoring system to sense completion of specific events and a signal transduction pathway relays this status to cell cycle system. The cell cycle is governed by transitions through phases that are irreversible due to these feedback signals in reaction networks [126]. 

In eukaryotes, cell cycle transitions in eukaryotes are driven by cyclin-dependent kinases (CDK). CDK are enzymes that regulate target proteins through phosphorylation that execute DNA replication and mitosis. A subset of CDK includes Cdk1 and Cdk2, the timing of cell cycle transitions is based on oscillations in cyclinB which binds to Cdk1. Cdk1 and Cdk2 form complexes with cyclins that are involved in timing regulation of the eukaryotic cell division cycle but are themselves regulated by oscillations in the phosphorylation of highly specific active binding sites and inhibitory binding sites by CKI (Cdk inhibitors) [127]. The G1/S transition is a toggle switch based on mutual inhibition of Cdk1-cyclinB and CKI while the S/G2 transition is an irreversible transition due to Cdk1-cyclinB activity. In the G2 phase, Cdk1-CycB activity is inhibited by phosphorylation of the tyrosine subunit of Cdk which is reversible. G2-M transition into mitosis is triggered by activation of the Cdk1-CycB complex by CycB activity exceeding a threshold—the threshold imposes reversibility., i.e., the G2/M transition is a toggle switch based on mutual activation of Cdk1-cyclinB and Cdc25 and mutual inhibition between CDK1-cyclinB and Wee1. The mitotic spindle forms in the M phase. In the M phase, chromosomes condense within the nucleus, the centrosomes divide and migrate to opposing sides of the nucleus. DNA damage during G2 induces CKI production and prevents passage through the checkpoint to M phase by tyrosine phosphorylation of the p34-cyclinB pathway while activating the p53 pathway [128,129]. The M/G1 transition is an oscillator based on negative feedback of Cdk1-cyclinB activating APC which activates Cdc20 which degrades cyclinB. M-G1 transition out of mitosis is triggered by repression of CDk1 by the proteolysis of CycB initiating cell division and the G1 phase. Proteolysis is crucial in maintaining low basal levels of regulatory proteins by recycling the proteins when tagged by the short polypeptide ubiquitin [130]. Ubiquitin is an essential protein in coordinating regulation of multiple simultaneous pathways by tagging some proteins for destruction (proteolysis) and modifying the function of other proteins [131]. Ubiquitin forms the basis of a histone code in which modification of histone proteins are translated into activation or silencing of gene transcription such as histone methylation. In G1, Cdk1-CycB activity is inhibited by CKI. G1-S transition to DNA replication is initiated by Cdk1-CycB complex activity. The eukaryotic cell cycle of Cdk levels may be modelled as a set of nonlinear ordinary differential equations [132,133]:

$$\frac{d\left[CycB\right]}{dt}=k_{1}-\left({k^{\prime }}_{2}+k^{\prime \prime }{}_{2}\left[Cdh1\right]\right)\left[CycB\right]$$

$$\frac{d\left[Cdh1\right]}{dt}=\frac{(k\prime_{3}+k^{\prime \prime }{}_{3}A\left(1-\left[Cdh1\right]\right)}{J_{3}+1-\left[Cdh1\right]}-\frac{k_{4}m\left[CycB\right]\left[Cdh1\right]}{J_{4}+\left[Cdh1\right]}$$

where [Chdh1], [CycB] = concentrations of Cdh1 and CycB, k = rate constants, J = Michaelis constants, and m = cell mass (G2/M checkpoint). This oscillatory system exhibits checkpoints corresponding to stable steady states that enforce time delays while phase transitions correspond to irreversible bifurcations into cell phases based on Chd1 and CycB levels [134]. The eukaryotic cell cycle also exhibits functional redundancy with several cyclins (A, B and E) and Cdks (1 and 2), e.g., CyclinB supports mitosis, CyclinE supports DNA and centrosome replication and CyclinA supports all three processes [135]. In fact, cell cycle is more complex with an additional four phosphorylation–dephosphorylation cycles—pre-MPF/MPF, Cdc25P/Cdc25, Wee1P/Wee1 and APCP-APC [136]. Cyclin combines with Cdk to form maturation promoting factor (MPF); anaphase promoting complex (APC) is activated by cyclic degradation; Cd25 and Wee1 regulate MPF. In *Xenopus* eggs, the cell replication cycle involves a number of Cdk complexes with nonlinear interactions generating emergent properties of multistable states, limit cycles and switches [137]. While CDKs are crucial to the regulation of the cell cycle, there is a contribution from the emergence of a cell cycle oscillator (repressilator) from a transcription factor network of three transcriptional repressor systems [138,139]. Biological information processing within the cell has extremely high complexity forming vast networks [140]. Although cell cycles may be mapped onto sequential processes of the lunar industrial ecology, it is questionable that the subtleties of real biological networks could be emulated in any such artificial metabolism. 

## 10. Biological Circuitry

**Figure 6:**
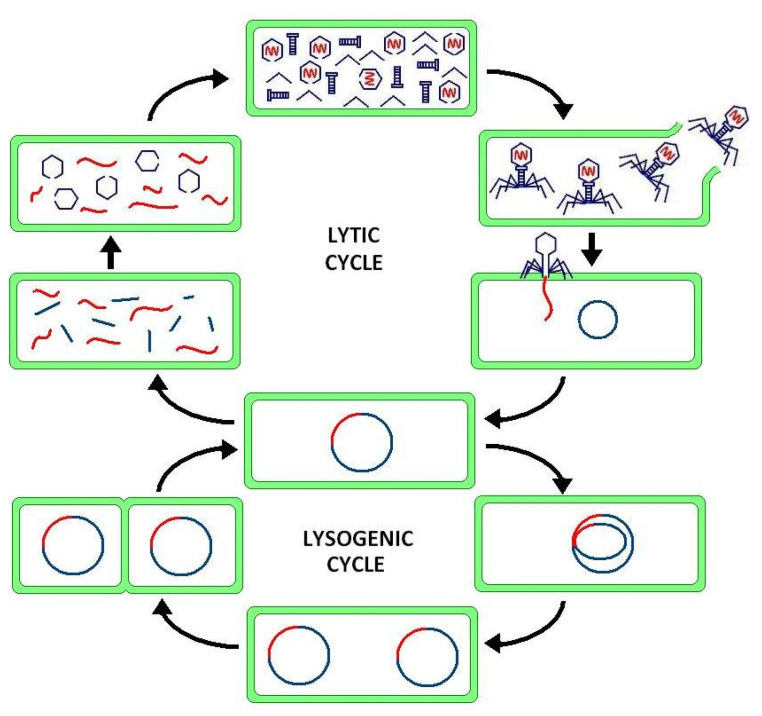
Lytic-lysogeny cycles (GNU Free Documentation License 1.2).

Cellular processes arise from the interconnections between genes and proteins forming the signalling pathways of GRN. However, genetic expression is subject to intrinsic noise in promoter, mRNA and protein activity and extrinsic noise from environmental cellular fluctuations which introduces phenotypic variation, e.g., in lysis-lysogeny switch [141,142] (Figure 6). The key to noise tolerance in all biological circuitry is feedback [143]. Negative feedback loops implement homeostasis or damped oscillation while positive feedback loops implement multistability (switching). Negative feedback is a noise reduction mechanism that low pass filters high frequency components. An example oscillatory transcription is the circadian rhythm of day-night cycles based on the phosphoform cycle mediated by coupling of a delayed negative feedback (morning transcription activates daytime transcription which activates nighttime transcription which represses morning transcription) and a repressilator (morning transcription represses nighttime transcription which represses daytime transcription which represses morning transcription) [144]. The bacterial cell cycle of *Caulobacter* has also been modelled as an oscillatory finite-state machine controlled by a few time-controlled master regulator proteins [145]—DnaA, CtrA, GcrA, and CcrM, the first two of which control DNA replication through DNA hemimethylation as the replication fork passes through those methylated encoding genes. One regulatory protein, CtrA, is involved in over 25% of bacterial cell cycle regulation of genetic transcription [146]. Genetic control circuits implement balancing between negative feedback loops to stabilise deviances in output signals (robustness) while positive feedback increases sensitivity to noisy input signals (adaptation). A combination of feedforward control to reject a large preset disturbances supplemented by integral feedback control for finer adaptive disturbance rejection offers a robust biochemical strategy despite its tendency to saturation instability [147]. An oscillation between negative and positive feedbacks is responsible for repetitive segmentation stripes in insect embryos due to differential gene expression. Negative feedback introduces instability with time delays. The toggle switch is a bistable on/off switch implemented through positive feedback whose stability is unaffected by time delays [148]. Positive feedback exploits stochastic noise to generate a stable response to perturbations, i.e., noise tolerant [149]. For example, bacteriophages can exist in one of two states within a bacterial cell—in the lytic state, the virus replicates itself and then lyses the bacterial cell to release the viral particles; in the lysogenic state, viral DNA integrates itself into the bacterial DNA. In this case, the repressor inhibits expression of other viruses acting as a switch implemented through a positive feedback loop. In a demonstration of design modularity, the toggle switch may be interfaced with different signalling pathways to impose a binary response [150]. 

Positive feedback may be implemented through double feedback loops: a double positive feedback loop involves two activators that activate each other for both ON or OFF; a double negative feedback loop involves two repressors that repress each other for ON/OFF or OFF/ON. Double feedback loops exhibit the properties of positive feedback loops but yield irreversible rather than reversible bistable (toggle switch) response [151]. In the double negative feedback loop, protein A (such as LacI) represses protein B (such as TetR) while protein B represses protein A. Irreversible bistability results with either A-on/B-off (TetR-off) or A-off/B-on (TetR on) but A and B cannot be in the same state. Any even number of negative feedback circuits yields bistability but any odd number of negative feedback circuits yields oscillatory response, e.g., three negative feedback circuits yields the repressilator (ring oscillator) that generates periodic oscillations in the concentrations of the three proteins—LacI protein represses the promoter for the tet gene, the TetR protein represses the promoter for the cI gene, and the CI protein represses the promoter for the lac gene. Robustness is a distinctive property of biological systems which ensures maintenance of biological function despite external or internal perturbations in which feedback and feedforward control play a central role [152,153]. Biological physiology tends to employ integrator-based controllers to ensure robust response to variations in environmental states but biological physiology is characterised by networks of interlinked processes that afford system robustness [154]. Robustness is enabled through hierarchical feedback control systems, especially integral feedback, which in biological organisms, is implemented through GRN [155]. Negative integral feedback provides robust adaptation while positive feedback amplifies stimuli to generate robust bistable response. 

Redundancy is another widely adopted approach to robustness in biological systems. Functional redundancy may be implemented through genetic buffering involving either duplicate genes (which rapidly diverge in functionality) or different genes with similar functionality. Modular redundancy (e.g., genetic duplication) imposes the cost of replicated modules while functional redundancy (e.g., phenotypic plasticity) employs different systems to perform the same function. Biological robustness affords evolvability and itself is afforded by evolution, e.g., genetic duplication is a mechanism for evolutionary innovation including functional redundancy such as anaerobic glycolysis and oxidative phosphorylation for ATP production [156]. Hox genes are an example of highly conserved genetic modularity for the development of multicellular body plans. Furthermore, transcriptional regulatory networks in bacteria have exhibited convergent evolution with independent evolution in different species [157]. Heat shock response in cells involves molecular heat shock chaperones as a universal genetic module under feedback control that provides robustness to heat stress by reversing protein unfolding [9]. We have addressed these factors of robustness elsewhere [158]. It is clear that there are strong correlations between biological control systems and electronic control systems. In unit chemical processors, sophisticated process control systems are required to monitor and control process throughput with both robust and adaptive behaviour. The lunar industrial ecology, as in biological systems, will involve complex feedback and feedforward control systems. 

Synthetic biology proposes to construct genetic circuits within biological cells to engineer complex information processing functions [159,160]. Synthetic biological circuits typically take hours to compute. A registry of genetically encoded standard biological parts (BioBricks) with well-defined transfer functions—such as sensors, logic gates and actuators—permits the construction of hierarchies of engineered devices and systems [161]. It is also possible to dynamically self-tune behaviour through the control of transcription in response to environmental sensing [162]. GRN may be employed as natural modules such as the lac operon to construct synthetic biology circuits such as toggle switches and ring oscillators (repressilator) [163]. In essence, signals are mapped onto GRN with computation performed through the rates of transcription factor binding to DNA [164]. A component library of logic gates may be constructed from a biochemical inverter based on transcription/translation repressor proteins [165]. Logic processing in genetic circuits involve promoters which recruit RNA polymerase to transcribe downstream genes—promoters are either activated or repressed by transcription factors. A biological NAND gate can be constructed from two parallel inverters connected to a downstream inverter. The implication function uses an inducer to bind the repressor. A genetically encoded universal NOR gate is more straightforward to implement than NAND [166]. A GRN implementing a biological half-adder has been constructed from an AND and an EXOR gate [167]. From these logic gates, the toggle switch and repressilator can be constructed. A genetic toggle switch implemented through two repressors and two promoters in a mutually inhibitory gene regulatory network offers a memory cell [168]. The genetic memory cell can potentially implement epigenetic inheritance. The repressilator comprises a daisy chain of promoter-repressor pairs to create a negative feedback loop. The Lac and Ntr systems were employed to construct multi-module genetic circuits with damped oscillatory or toggle switch behaviours [169]. An activator module was common to both circuits but the oscillator also incorporated a repressor module. A transient pulse-generating network integrated positive and negative regulation of gene expression through a feedforward circuit in response to a long-lasting signal [170]. An engineered gene circuit can implement a synchronised oscillation (clock) for coordinating activity through a multicellular population based on quorum sensing when that population is large enough [171,172]. From these basic functions, more complex genetic circuits can be constructed [173]. 

The more sophisticated cell protein signalling networks of eukaryotes have a modular regulatory organisation which offers the prospect for rewiring [174]. Evolutionary programming is one approach to evolving biological networks in silico implementing simple filters from modules based on Michaelis–Menten reaction kinetics in protein/protein networks and Hill equations in gene/protein networks [175]. However, challenges in predicting genetic circuit performance (e.g., molecular stability, binding strength, and rate constants) requires the employment of in vivo directed evolution rather than model-based evolutionary programming to construct more complex circuits [176]. Although we have focussed on transcription factors, biological genetic regulation is multi-faceted including non-transcriptional regulation [177], e.g., trans-acting RNA molecules such as microRNAs rather than proteins. RNA regulation is more stable but with slower response than transcription factor regulation in synthetic feedback control circuits [178]. RNA-only post-transcriptional ribocomputing circuits of combinatorial logic gates implemented in bacteria offer high predictability of base-pairing rules to permit the use of in silico modelling [179]. However, constructing engineered biological systems remains challenging due to replication errors [180]. This makes synthetic biological cells unstable. Synthetic circuits are lost over accumulated cell doublings but there are a few strategies to minimise synthetic circuit loss [181]—minimise repeated sequences which are rapidly excised; decrease expression levels and so metabolic load; design inducers rather than promoters to increase metabolic load; reduce genome size by eliminating insertion sequences to reduce mutation rate. However, some of these strategies are difficult to implement. A further possibility is to supplement synthetic biological circuits with in silico circuits such as employing an in silico feedback control algorithm outside of the cell to respond to an external signal [182]. Small modules with basic functionalities such as oscillators or switches have been evolved in silico for the formation of larger genetic regulatory networks [183]. A further step in hybridising electronic signalling between biological and nonbiological systems is to introduce a biocompatible electron transfer conduit from the cell interior through the insulating cell membrane to extracellular electron acceptors [184]. A chemical kinetic implementation of a McCulloch–Pitts neuron has been achieved through a cyclic enzyme reaction in which high/low concentrations were dependent on the concentration of a catalyst through either excitatory or inhibitory enzymes [185]. The McCulloch–Pitts neuron can construct AND, OR, NAND and NOR gates. An oscillating catalyst imposed a discrete timing mechanism. This was extended to a network of McCulloch–Pitts neurons with chemically weighted connections to form a variety of finite-state machines including a binary adder and a stack memory [186]. In principle, a chemical universal Turing machine could be constructed from two chemical memory stacks and a clocked neural network. 

## 11. Conclusions

Our lunar industrial ecology represents a sustainable approach to ISRU which feeds into a manufacturing system. Although multiple machines that must be coordinated are characteristic of manufacturing, this is not so in chemical processing plants. The lunar industrial ecology, however, involves multiple chemical processors that must be coordinated with extensive recycling loops. The design and architecture of manufacturing factories may be applied to the lunar industrial ecology and these approaches have been reviewed. Although these approaches are appropriate throughout the processing chain from mining to final product, a central facet of any ISRU system will involve exploration of the planetary environment and the acquisition of physical resources [187]. This will require complex strategies involving coordination of multiple roving robots. We believe that the holonic architecture is most appropriate for organising the lunar industrial ecology. Biological systems through the use of inducer-repressor systems implement a system of double negative logic rather than positive logic adopted in engineering. This generates subtleties to biological systems. Within this framework, GRNs appear promising but are complex—Bayesian networks offer an approach for emulating GRN behaviours with certain provisos. More encouragingly, there are direct relationships between biological control systems and those employed in engineering, and indeed, synthetic biology demonstrates that engineered systems can be implemented in biological cells. They also provide the basis for checkpointing in the cell cycle. Although this addresses the basic mechanisms of metabolic control, the application of metabolic networks, however, may be more promising. An artificial chemistry with self-maintaining chemical reactions represented as graphical rewrite rules has been subjected to dynamic programming (energy) constraints with fitness determined by metabolic yield to simulate the evolution of simple metabolic networks [188]. Genetic regulatory networks have been evolved in silico using genetic programs acting on a set of master reactions (translation, transcription, dimerization and degradation) with oscillatory dynamics [189]. The expression dynamics of an artificial genome may be modelled graphically as a Boolean network encoded through promoters with evolution implemented through a genetic algorithm selecting according to gene number and connectivity [190]. This simulates the evolution of a genetic regulatory network indicating that the construction of more complex networks is more rapid from simpler cyclic network precursors. This correlates with the synthetic evolution of small genetic regulatory networks to bootstrap more complex, modular genetic regulatory networks [191]. A technique of aggressive pruning in directed synthetic biological evolution generated genetic regulatory networks for a bistable toggle switch and an oscillatory circuit which were used as modules to evolve more complex genetic regulatory networks. Engineered-by-design circuits are much noisier and unreliable than such evolved circuits due to stringent selection by the fitness function. The introduction of ontogenetic development into an artificial evolutionary system can evolve modular genetic regulatory networks with low pleiotropy [192]. Phenotypic novelty has been demonstrated in simulated evolution of multicellular organisms to be a product of diversity of evolved developmental gene regulation modelled as Boolean networks rather than structural genes [101]. Genetic regulatory networks in bacteria appear to have evolved in much the same fashion through the emergence of hierarchical modules with increasing complexity [192]. Novel regulatory pathways arose rarely through horizontal gene transfer with traditional mutational mechanisms—duplication, point mutation and inversion—providing the main engine of evolutionary reorganisation of regulatory pathways. Horizontal gene transfer is enabled through a library of genes (metagenome of diverse gene cassettes) in bacterial communities that generate new pathway modules. Far more frequent is the rearrangement of the “wiring” of more complex functions from existing modules, e.g., CtrA protein is a master regulator in cell cycles but it is applied to different functions across species. The bacterial flagellum has common features of design and hierarchical assembly but mechanisms for integrating flagella into bacterial cells varies across species, e.g., distribution of flagella. The evolution of bacterial genetic regulatory networks appears to be an adaptive response to dynamically changing environmental stresses. These findings suggest that perhaps a promising approach might be to adopt genetic algorithms or genetic programs to evolve genetic regulatory networks for the lunar industrial ecology in a hierarchical manner. This remains to be explored. Nevertheless, it seems reasonable that some aspects of biological control systems can be implemented in engineered systems. However, the high complexity of GRNs and difficulties in modelling and prediction of their behaviours render them an unlikely biomimetic candidate for engineering implementation in the near term unless evolutionary methods are explored. 

## Figures and Tables

**Table 1:** List of desired materials (demandite) based on functional requirements for a generic spacecraft.

Functionality	Lunar-Derived Material
Tensile structures	Wrought iron Aluminium
Compressive structures	Cast iron
Elastic structures	Steel springs/flexures Silicone elastomers
Hard structures	Alumina
Thermal conductor straps	Fernico (e.g., kovar) Nickel Aluminum
Thermal insulation	Glass (SiO_2_ fibre) Ceramics such as SiO_2_
High thermal tolerance	Tungsten Alumina
Electrical conduction wire	Fernico (e.g., kovar) Nickel Aluminium
Electrical insulation	Glass Ceramics (SiO_2_, Al_2_O_3_and TiO_2_) Silicone plastics Silicon steel for motors
Active electronics devices (vacuum tubes)	Kovar Nickel Tungsten Fused silica glass
Magnetic materials	Ferrite Silicon steel Permalloy
Sensory transducers	Resistance wire Quartz Selenium
Optical structures	Polished nickel/aluminium Fused silica glass
Lubricants	Silicone oils Water
Combustible fuels	Oxygen Hydrogen

**Table 2:** Lac operon as a Boolean “if…then…” operator.

Allolactose	Repressor	Operon
0	0	0
0	1	1
1	0	0
1	1	0
